# Cigarette smoke extract and heated tobacco products promote ferritin cleavage and iron accumulation in human corneal epithelial cells

**DOI:** 10.1038/s41598-021-97956-3

**Published:** 2021-09-17

**Authors:** Wataru Otsu, Kodai Ishida, Naoki Chinen, Shinsuke Nakamura, Masamitsu Shimazawa, Hideshi Tsusaki, Hideaki Hara

**Affiliations:** 1 Department of Biomedical Research Laboratory, Gifu Pharmaceutical University, 1-25-4 Daigaku-nishi, Gifu, 501-1196 Japan; 2 Molecular Pharmacology, Department of Biofunctional Evaluation, Gifu Pharmaceutical University, 1-25-4 Daigaku-nishi, Gifu, 501-1196 Japan

**Keywords:** Stress signalling, Autophagy

## Abstract

The cornea is directly exposed to cigarette smoke, and smoking is a risk factor for several corneal diseases including dry eye syndrome. Currently, heated tobacco products (HTPs) are widely used as substitutes for cigarette smoking around the world. In the present study, we investigated the molecular mechanism(s) leading to cellular injury induced by cigarette smoke extract (CSE) or HTPs. Exposure to CSE perturbed the formation of tight junctions, leading to an increase in cell volume, a decrease in transepithelial electrical resistance (TER) in the human corneal epithelial cell-transformed (HCE-T) cell line. Moreover, CSE exposure induced both lipid peroxidation and ferrous [Fe(II)] ion accumulation in autolysosomal compartments. Interestingly, a cleaved form of ferritin appeared when HCE-T cells were incubated with CSE. This aberrant ferritin processing was suppressed by treatment with autophagy inhibitors. Furthermore, the CSE-induced cell death was suppressed by either ferrostatin-1 or deferoxamine (DFO). CSE exposure also promoted the expression of cytokines whereas DFO treatment inhibited the CSE-induced expression of these cytokines. Exposure to HTPs also induced both HCE-T cell death and cleaved ferritin accumulation in a concentration- and time-dependent manner. These results indicated that CSE or HTPs activated the ferroptosis signaling pathway, which contributed to corneal epithelial cell injury.

## Introduction

The corneal epithelium is the outermost layer of the cornea, which is exposed to environmental factors including light, pathogens, and chemicals. It is composed of stratified squamous epithelial cells located on top of a single layer of basal cells. The corneal epithelium is constantly renewed; the proliferation of corneal epithelial precursors and the development of the corneal epithelium from the basal cell layer are essential for maintaining the corneal layers. The corneal and conjunctival epithelial cells secrete several types of mucins, which are a family of heavily *O*-glycosylated glycoproteins with high molecular weight. Mucins are the main structural components of the mucin layer, which is the innermost layer of the tear film^1^.

Clinical surveys revealed that smoking affects the tear film^2^ and experimental analyses with animal models showed that exposure to mainstream cigarette smoke damaged the cornea and lacrimal glands^3^. While smoking can contribute to dry eye syndrome, it is also associated with many ocular diseases including age-related macular degeneration^4^. Since the eyes are directly exposed to sidestream and second-hand smoke, the cornea is especially sensitive to these environmental factors. Heated tobacco products (HTPs), also referred to as heat-not-burn tobacco, are widely used as substitutes for cigarettes because they have no sidestream smoke emission and are believed to be less harmful. Although mainstream HTPs contain less hazardous compounds compared to those found in conventional combustion cigarettes, HTPs contain toxic compounds including nicotine^5^. Importantly, the effects of HTPs on the corneal epithelium cell death pathway have not been thoroughly characterized.

Cigarette smoke contains over 7,000 constituents including tar, nicotine, and free radicals^6^. Chronic cigarette smoke exposure induces an aberrant inflammatory process, leading to the pathogenesis of pulmonary disorders including chronic obstructive pulmonary disease (COPD)^7^. Cigarette smoke contains particulate matters including iron, which induces oxidative stress by altering iron homeostasis^8^. Iron plays an important role in the cellular defense against oxidative stress. Ferroptosis, an iron-dependent, non-apoptotic programmed cell death pathway, is characterized by phospholipid peroxidation though the iron-mediated Fenton reaction^9^. Recently, it has been reported that ferroptosis is involved in cigarette smoke-induced pulmonary epithelial cell damage^10^. Under iron-starved conditions, ferritin (a ubiquitously expressed protein that stores intracellular iron) is degraded via the autophagic pathway. This results in the release of the stored ferric iron, which is referred to as ferritinophagy^11^. However, it remains unknown whether ferroptosis and/or ferritinophagy are involved in cigarette smoke-induced corneal damage.

Here, we investigated the effects of cigarette smoke extract (CSE) and HTPs on the corneal epithelium using an in-vitro cell culture system. CSE treatment perturbed the structure and function of the epithelial barrier, which were remediated by treatment with *N*-acetylcysteine (NAC). CSE exposure induced intracellular lipid peroxidation and iron accumulation. Additionally, CSE exposure increased the levels of a cleaved form of ferritin. Treatment with the ferroptosis inhibitor, ferrostatin-1 (Fer-1) or the iron chelator, deferoxamine (DFO), protected HCE-T cells against CSE-induced cell death. Interestingly, HTPs also induced both cell death and cleaved ferritin accumulation in HCE-T cells. These results provided insights into the mechanism of corneal epithelium damage induced by CSE or HTPs.

## Results

### The effects of NAC on CSE-induced cellular damage and epithelial barrier dysfunction in HCE-T cells

In order to investigate the effects of CSE exposure on the corneal epithelium, we used HCE-T cells as an in-vitro cell culture model. Exposure of HCE-T cells to CSE for 24 h induced cell death and the decrease in cell viability occurred in a concentration-dependent manner (Supplementary Fig. 1). As reported previously^12^, 5% CSE exposure significantly increased the cell death ratio in HCE-T cells (Supplementary Fig. 1B,C). In addition, NAC, a precursor of reduced glutathione (GSH), suppressed CSE-induced cell death (Supplementary Fig. 1B,C). The reduced cell viability induced by CSE exposure was also ameliorated in the presence of NAC (Supplementary Fig. 1D), which suggested that oxidative stress plays a role in CSE-induced cell damage.

**Figure 1:**
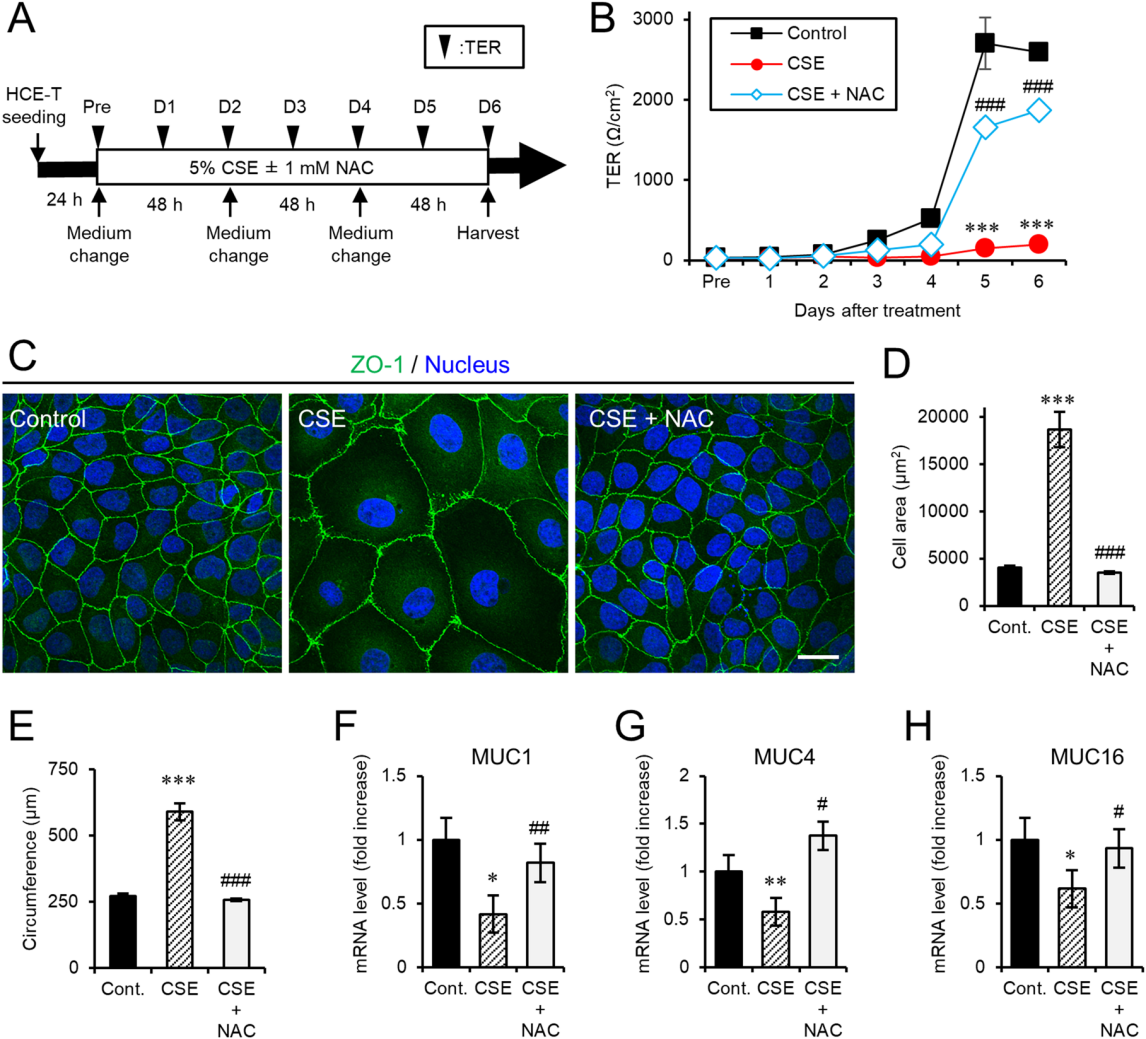
NAC treatment ameliorated epithelial barrier dysfunction of HCE-T cells exposed to CSE. (**A**) The schematic diagram depicts the treatment time courses and the examinations. The cells were harvested at day (D) 6 for immunofluorescence staining or qRT-PCR. (**B**) The graph shows the TER measurements before (pre) and after CSE exposure. (**C**) Representative projection view immunostaining (ZO-1, green; Hoechst 33342, blue) images of HCE-T cells at day 6 after CSE exposure. Bar = 100 µm. (**D**, **E**) The quantification of the immunostaining shown in C for the cell area (**D**) and circumference (**E**). (**F**–**H**) Quantitative PCR analyses of MUC1 (**F**), MUC4 (**G**), and MUC16 (**H**) at day 6 after CSE exposure. The data are represented as the mean ± SEM (n = 6). *P < 0.05, **P < 0.005, ***P < 0.001, Student’s *t*-test vs. Control (Cont); ^#^*P* < 0.05, ^##^*P *< 0.005, ^###^*P* < 0.001, Student’s *t*-test vs. CSE.

Next, we investigated the effects of CSE on epithelial barrier function in polarized HCE-T cells. To this end, transepithelial electrical resistance (TER) measurements were obtained and immunostaining of tight junctions was conducted in the presence or absence of 5% CSE (Fig. 1A). In cells that were not exposed to CSE, the TER value gradually increased and reached a value greater than 2,500 Ω/cm^2^ at 5 days after spreading on a filter membrane (Fig. 1B). In contrast, the TER values from cells exposed to CSE remained significantly lower than the control cells lacking CSE exposure (Fig. 1B). While the cell–cell adhesion of polarized HCE-T cells lacking CSE exposure exhibited a typical hexagonal pattern at day 6 after the medium change, this structure was completely disrupted in the cells cultured with 5% CSE (Fig. 1C). Quantification of the cell area and circumference revealed that both of these parameters were significantly increased by CSE exposure (Fig. 1D,E). Furthermore, the expression levels of several mucins (MUC1, MUC4, and MUC16) were decreased when cells were incubated with 5% CSE (Fig. 1F–H). On the other hand, NAC treatment increased the TER values (Fig. 1B), preserved the tight junction structure (Fig. 1C–E), and stimulated the expression of mucins to control levels (Fig. 1F–H). These results indicated that CSE exposure leads to epithelial barrier dysfunction in corneal epithelial cells, and the antioxidant defense system mediated by GSH can attenuate these defects associated with epithelial barrier dysfunction.

### CSE-induced lipid peroxidation and iron redistribution in HCE-T cells

**Figure 2:**
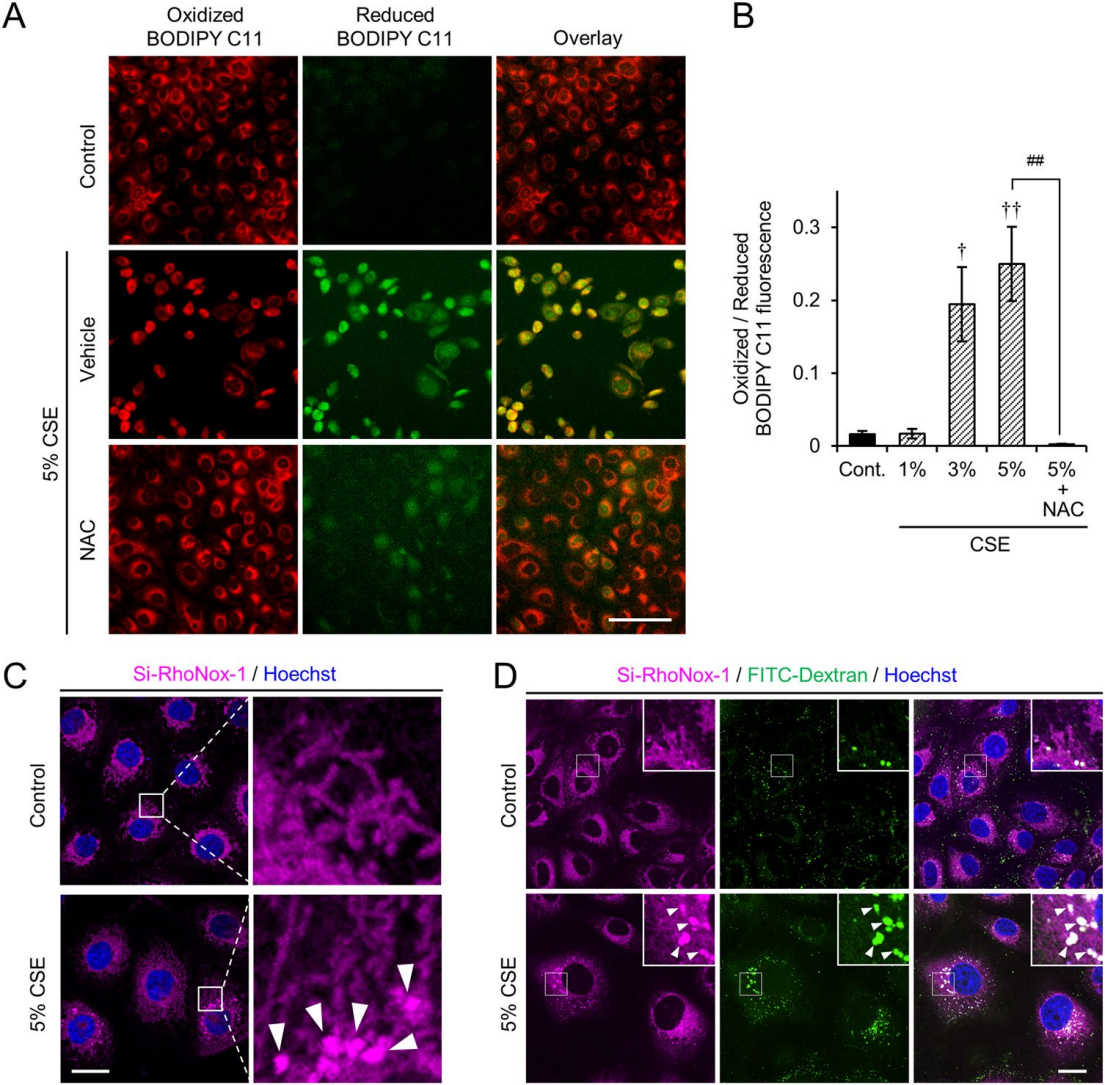
Exposure of HCE-T cells to CSE resulted in lipid peroxidation and autolysosomal iron accumulation. (**A**) Representative images of BODIPY 581/591 C11 (BODIPY C11) fluorescence in live HCE-T cells incubated without (control) or with 5% CSE in the presence of vehicle (PBS) or 1 mM NAC for 24 h. (**B**) The fluorescence intensity ratios for oxidized (green) and reduced (red) BODIPY C11. The data are represented as the mean ± SEM (n = 6). ^†^*P* < 0.05, ^††^*P* < 0.005, Dunnett’s test vs. Control (Cont); ^##^*P* < 0.005, Student’s *t*-test vs. 5% CSE. (**C**) Representative images of iron distribution in live HCE-T cells following CSE exposure. The cells were incubated with Si-RhoNox-1 (magenta) and Hoechst 33342 (blue), 30 min before recording. The enlarged views of the boxed regions are shown on the right. (**D**) Intracellular distributions of Si-RhoNox-1 in FITC-dextran (green) and Hoechst 33342 (blue) stained HCE-T cells with or without CSE exposure. The enlarged views of the boxed regions are shown at the upper right corners. The arrowheads indicate iron accumulation at the perinuclear regions. Bar = 100 µm (**A**), 20 µm (**C**,**D**).

CSE contains numerous chemicals that induce the production of reactive oxygen species (ROS), which evoke lipid oxidation and activate cell death signaling pathways^6^. We traced lipid peroxidation using the fluorescent probe, BODIPY 581/591 C11. As expected, the oxidized BODIPY signal was significantly increased in cells exposed to CSE, and it was attenuated by treatment with NAC (Fig. 2A,B). Labile iron pools initiate lipid peroxidation by generating free radical species via the Fenton reaction^13^. Therefore, we examined the iron distribution in live cells using a bivalent iron tracer^14^. Interestingly, the iron tracer signal, Si-RhoNox-1, exhibited a perinuclear globular distribution pattern following CSE exposure (Fig. 2C), which colocalized with uptaken fluorescein isothiocyanate (FITC)-dextran (Fig. 2D). This observation suggested that CSE induced iron accumulation in endo- and/or autolysosomal compartments.

### Intracellular ferritin degradation in CSE-treated HCE-T cells

**Figure 3:**
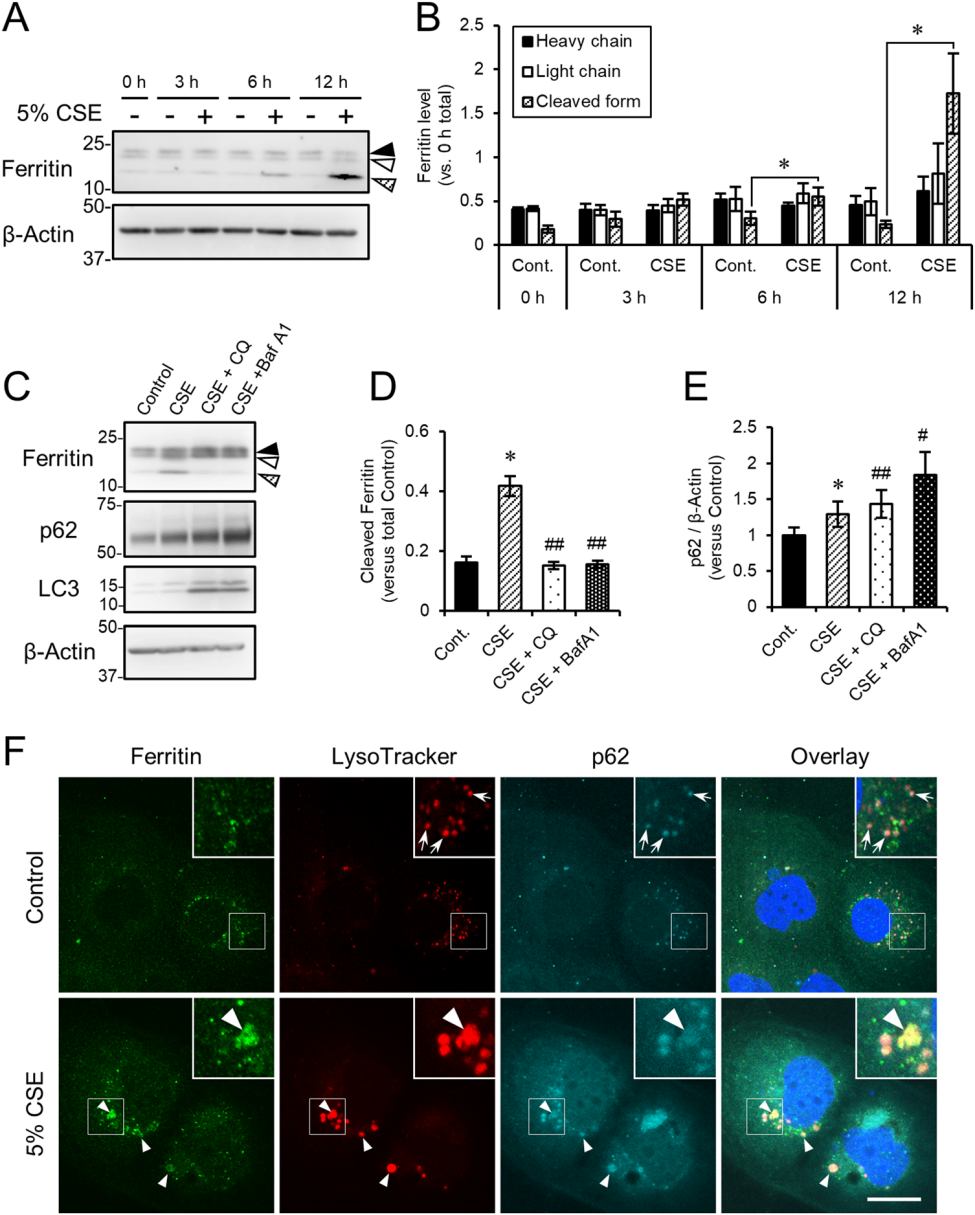
Exposure of HCE-T cells to CSE resulted in aberrant autophagic ferritin processing and accumulation of a cleaved form of ferritin. (**A**,**B**) HCE-T cells were exposed to CSE for the indicated times. Protein lysates were then analyzed by immunoblotting using antibodies directed against ferritin and β-actin (loading control). (**B**) Quantitation of the fold increase of each form of ferritin (heavy chain, black arrowheads; light chain, white arrowheads; cleaved form, arrowheads filled with diagonal lines) after exposure to CSE. (**C**–**E**) HCE-T cells were incubated without (control) or with 5% CSE in the absence or presence of either 25 µM chloroquine (CQ) or 10 nM bafilomycin A1 (BafA1) for 24 h. Protein lysates were then analyzed by immunoblotting using the indicated antibodies. (**D**,**E**) show the quantification of ferritin (the cleaved form) and p62, respectively, both normalized to β-actin. (**F**) Representative images of intracellular ferritin distribution in HCE-T cells with or without 5% CSE exposure for 12 h. The cells were stained with anti-ferritin antibody (green), LysoTracker Red (red), anti-p62 antibody (cyan), and Hoechst 33342 (blue). The boxed regions are enlarged at the upper right corners. The arrows represent p62 and LysoTracker Red double positive organelles, which comprise autolysosomes. The arrowheads show the autolysosomes that contain ferritin. Bar = 20 µm. The data are represented as the mean ± SEM (n = 6). *P < 0.05, Student’s *t*-test vs. Control (Cont); ^#^*P* < 0.05, ^##^*P* < 0.005, Student’s *t*-test vs. CSE.

Ferritin is a protein complex that stores intracellular iron. Ferritin is composed of two types of chains, the L- and H-chains. We used the anti-ferritin antibody that detects both L- and H-chains of human ferritin. In the control cells, ferritin signals appeared as two bands (Fig. 3A). While CSE treatment affected neither L- nor H-chain signals (Fig. 3A), an additional lower band at the position of approximately 10 kDa appeared at 6 h following CSE exposure (Fig. 3A). This faster migrating band was increased after CSE exposure in a time-dependent manner (Fig. 3A,B). This band also disappeared when cells were treated with the autophagic inhibitors, chloroquine (CQ) and bafilomycin A1 (BafA1), which suggested that the lowest ferritin band represented a cleaved product processed by autophagic degradation (Fig. 3C,D). Interestingly, CSE treatment significantly increased the expression of p62 (Fig. 3E). Concomitantly, immunostaining revealed that the intracellular ferritin signals were observed as perinuclear particles, which also contained p62 in the compartments stained with LysoTracker Red (Fig. 3F). Taken together, we concluded that CSE exposure accelerated the degradation of ferritin through autophagy, resulting in iron accumulation and lipid peroxidation.

### The protective effects of Fer-1 and DFO against CSE-induced HCE-T cell injury

**Figure 4:**
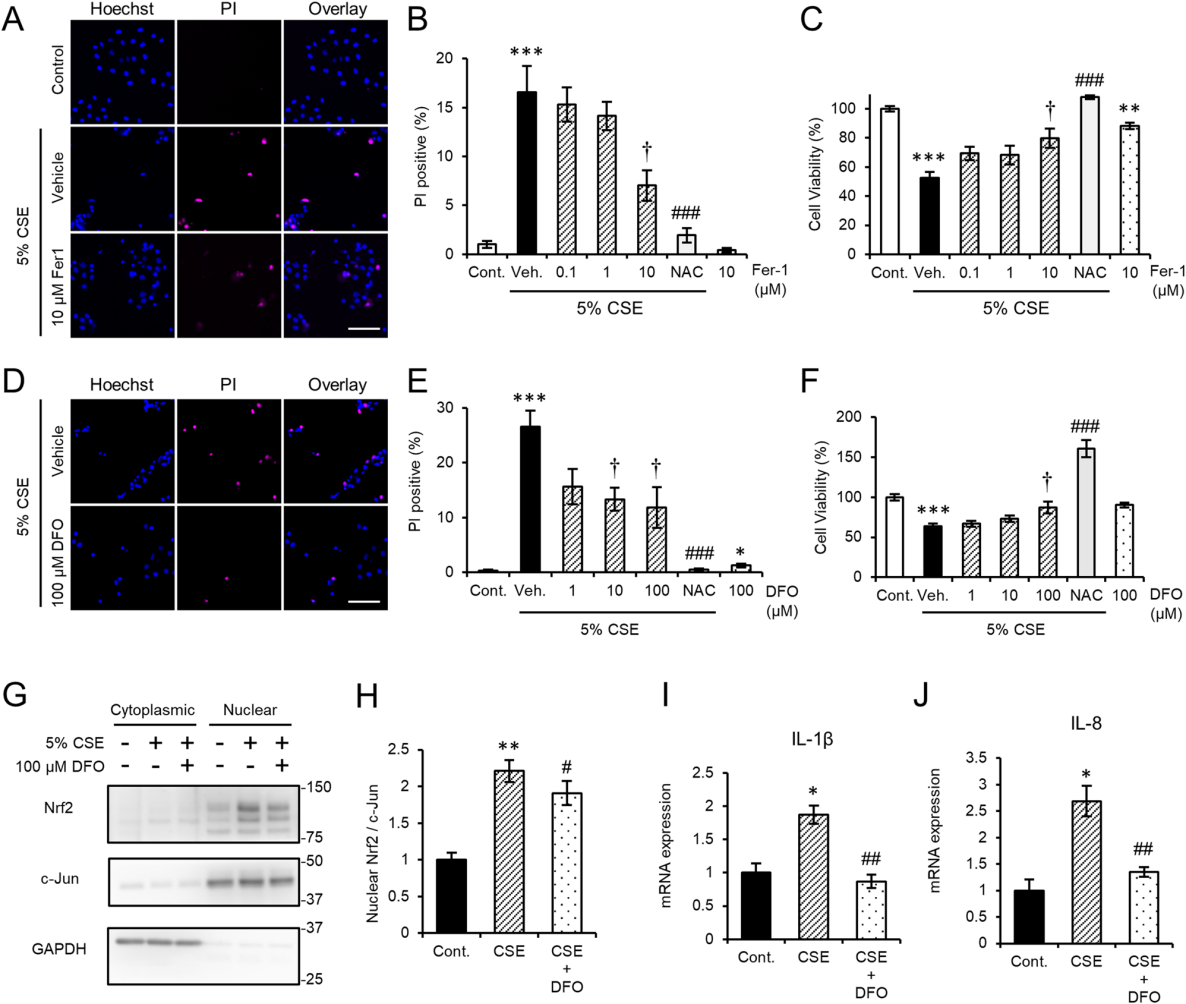
Fer-1 and DFO have protective effects against CSE-induced HCE-T cell damage. (**A**,**D**) Representative images of Propidium Iodide (PI, magenta) and Hoechst 33342 (blue) staining after CSE exposure in the presence or absence of Fer-1 (**A**) or DFO (**D**). Bars = 100 µm. (**B**,**E**) The bars show the PI-positive cell ratios. (**C**,**F**) Cell viability was measured with a Cell Counting Kit-8 (CCK-8) assay. (**G**,**H**) Subcellular fractionation showing the nuclear Nrf2 levels in HCE-T cells exposed to 5% CSE with or without 100 µM DFO treatment for 6 h. (**H**) Quantification of the nuclear Nrf2 levels shown in (**G**). The Nrf2 signals were normalized to the c-Jun signals. (**I**,**J**) IL-1β (**I**) and IL-8 (**J**) mRNA levels in DFO-treated HCE-T cells exposed to CSE. The data are represented as the mean ± SEM (n = 6). *P < 0.05, **P < 0.005, ***P < 0.001, Student’s *t*-test vs. Control (Cont); ^†^*P* < 0.05, Dunnett’s test vs. Vehicle (Veh); ^#^*P* < 0.05, ^##^*P* < 0.005, ^###^*P* < 0.001, Student’s *t*-test vs. Vehicle (Veh) or CSE.

Ferroptosis is defined as iron-dependent programmed cell death characterized by the accumulation of lipid peroxides^9^. Fer-1, a selective inhibitor of ferroptosis, was discovered by chemical library screening as a small molecule that prevented erastin-induced ferroptosis^9^. In order to investigate the involvement of ferroptosis in CSE-induced HCE-T cell injury, we monitored the effects of Fer-1 against HCE-T cell damage. We found that Fer-1 (10 µM) significantly reduced CSE-induced cell death (Fig. 4A,B) and ameliorated the reduction in cell viability induced by CSE (Fig. 4C). Furthermore, the iron chelator, DFO, significantly reduced the CSE-induced increase in the cell death ratio (Fig. 4D,E) and ameliorated the CSE-induced reduction in cell viability (Fig. 4F). Both effects mediated by DFO occurred in a concentration-dependent manner (Fig. 4E,F). Nuclear factor erythroid 2-related factor 2 (Nrf2), which is responsible for the cellular defense against oxidative stress, was increased in the nuclear fraction of CSE-treated HCE-T cells (Fig. 4G,H). In contrast, DFO treatment partially blocked CSE-mediated nuclear transport of Nrf2 (Fig. 4G,H), which indicated that DFO ameliorated the oxidative stress induced by CSE. In addition, in HCE-T cells exposed to CSE, DFO treatment suppressed the CSE-induced expression of various cytokines including IL-1β and IL-8 (Fig. 4I,J). Collectively, these results indicated that CSE can induce inflammatory cytokine secretion in corneal epithelial cells and intracellular iron plays a key role in its signaling pathway.

### Insights into the molecular mechanism of HCE-T cell damage induced by HTPs

**Figure 5:**
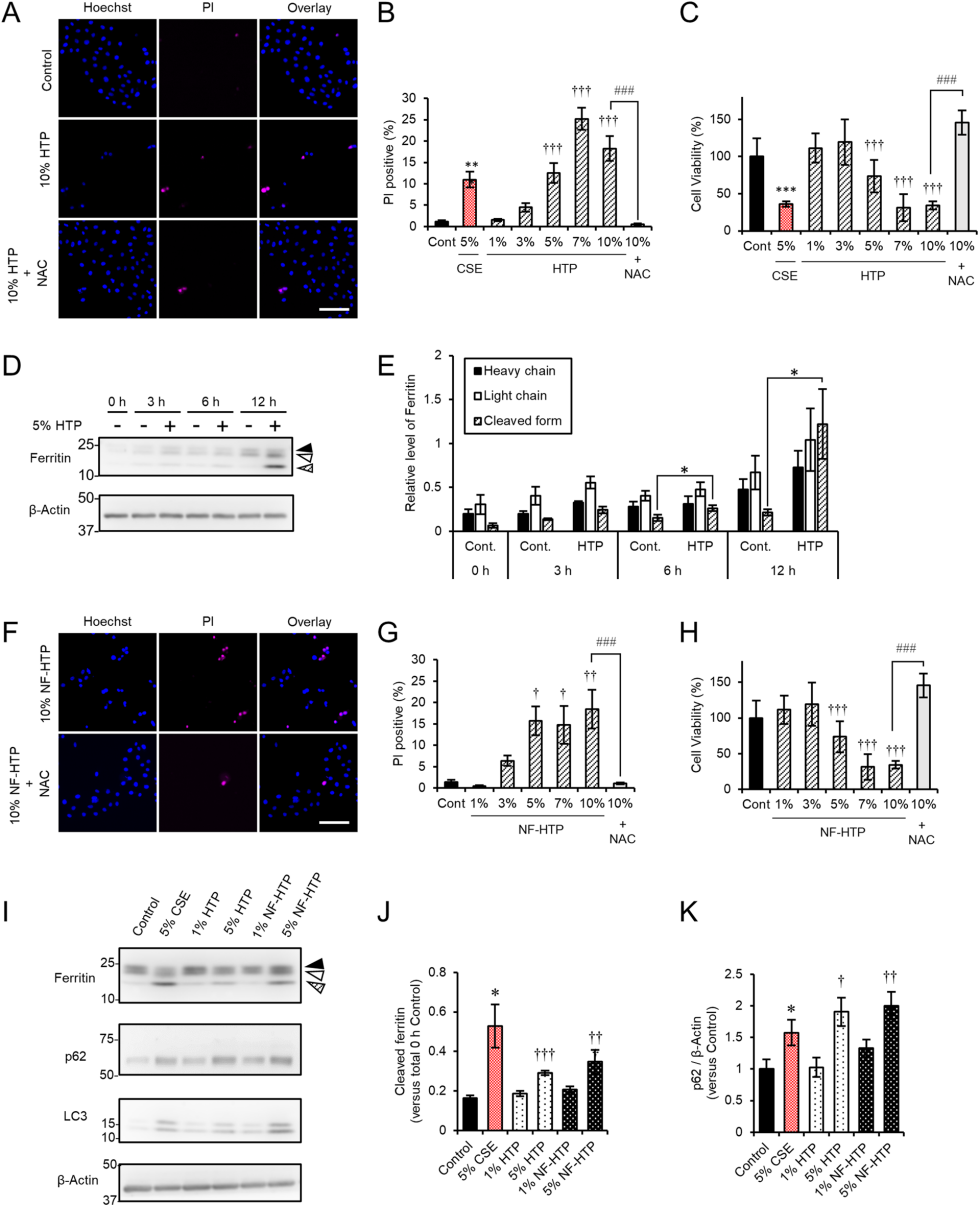
Exposure of HCE-T cells to HTPs induced cell death and accelerated ferritin cleavage. (**A**) Representative images of PI (magenta) and Hoechst 33342 (blue) staining after exposure to HTPs in the presence or absence of 1 mM NAC. (**B**) The bars show the PI-positive cell ratios. (**C**) Cell viability was measured with a CCK-8 assay. (**D**) Immunoblotting of HCE-T cell extracts exposed to HTPs. (**E**) Quantitation of the fold increase of each form of ferritin (heavy chain, black arrowheads; light chain, white arrowheads; cleaved form, arrowheads filled with diagonal lines) after exposure to HTPs. (**F**) Representative images of PI (magenta) and Hoechst 33342 (blue) staining after exposure to nicotine-free HTPs (NF-HTPs). (**G**) The bars show the PI-positive cell ratios. (**H**) Cell viability was measured with a CCK-8 assay. (**I**–**K**) Immunoblotting of extracts derived from HCE-T cells incubated without (control) or with either CSE (5%), HTPs (1 or 5%), or NF-HTPs (1 or 5%) for 24 h was conducted using the indicated antibodies. (**J**,**K**) show the cleaved form of ferritin and p62 (both normalized to β-actin), respectively. Bars = 100 µm (**A**,**F**). The data are represented as the mean ± SEM (n = 6). *P < 0.05, **P < 0.005, ***P < 0.001, Student’s *t*-test vs. Control (Cont); ^†^*P* < 0.05, ^††^*P* < 0.005, ^†††^*P* < 0.001, Dunnett’s test vs. Control (Cont); ^###^*P* < 0.001, Student’s *t*-test vs. 10% HTPs or NF-HTPs.

Recently, more and more HTPs are on the market as a substitute for cigarettes. These new generation devices heat tobacco leaves electrically, and the products from this low temperature non-burning heating system is thought to be less harmful. However, the effects of HTPs on the corneal epithelium have not been well-characterized. Herein, we found that HTPs induced HCE-T cellular damage with a similar efficacy as CSE (Fig. 5A–C). The cell damage induced by HTPs was also rescued by NAC treatment (Fig. 5A–C). Similar to CSE, HTPs also induced the generation of a cleaved form of ferritin in a time-dependent manner (Fig. 5D,E). Tobacco leaves contain nicotine, which is a toxic and addictive chemical compound^6^ and nicotine-free HTPs (NF-HTPs) are used for addiction treatment. Interestingly, exposure of HCE-T cells to NF-HTPs increased cell death (Fig. 5F,G) and suppressed cell viability (Fig. 5H), similar to the effects of HTPs. In addition, NAC treatment ameliorated the cellular damage induced by NF-HTPs (Fig. 5F–H). Moreover, ferritin cleavage was stimulated by treatment with either HTPs or NF-HTPs in a concentration-dependent manner (Fig. 5I,J). Furthermore, the levels of p62 were increased when cells were treated with either HTPs or NF-HTPs (Fig. 5I,K). These data indicated that the effects of HTPs on ferritin cleavage and HCE-T cell death were tar- and nicotine-independent.

**Figure 6:**
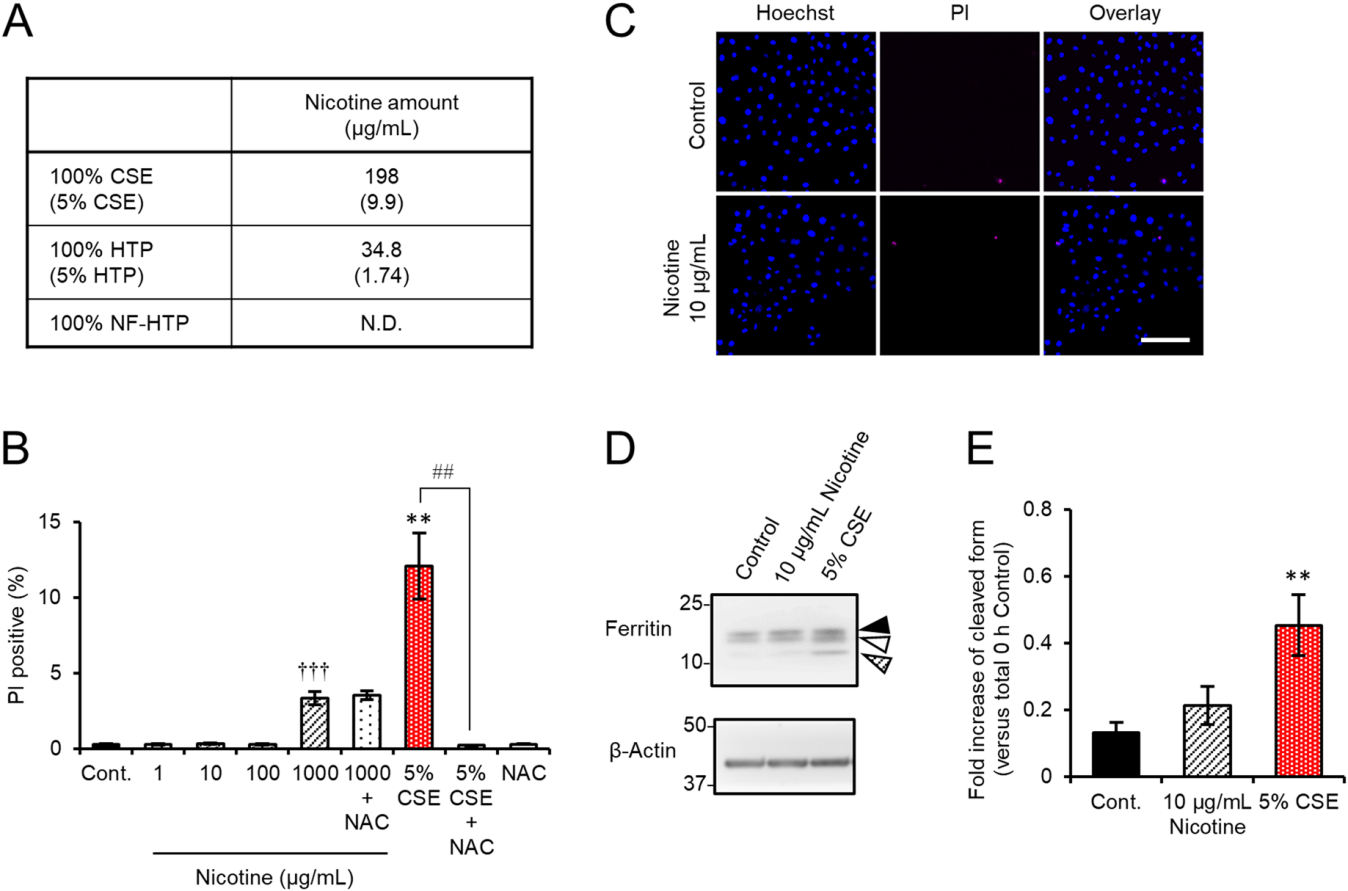
Neither cell death nor ferritin defect appeared in HCE-T cells treated with nicotine equivalent to 5% CSE. (**A**) Nicotine amount of CSE, HTP and NF-HTP used in the present study. (**B**,**C**) Results for cell death assay after exposure to nicotine (1, 10, 100, or 1,000 µg/mL) with or without 1 mM NAC. The quantification for the PI-positive cell ratios (**B**). Representative images of PI (magenta) and Hoechst 33342 (blue) staining (**C**). Bar = 100 µm. (**D**,**E**) Immunoblotting of extracts derived from HCE-T cells incubated without (control) or with either CSE (5%), or nicotine (10 µg/mL). (**E**) show the cleaved form of ferritin normalized to β-actin. Data represent the mean ± SEM (n = 6, **P < 0.005, Student’s *t*-test vs. Control; ^†††^*P* < 0.001, Dunnett’s test vs. Control; ^##^*P* < 0.005, Student’s *t*-test vs. 5% CSE).

Finally, we examined whether nicotine induced cell damage in HCE-T cells. The nicotine concentration of 100% CSE and 100% HTP used in the present study are 198 µg/mL and 34.8 µg/mL, respectively (Fig. 6A). The treatment of 1,000 µg/mL of nicotine alone caused cell death at 3.34 ± 0.43%, which is considerably lower than the one of 5% CSE (Fig. 6B). Neither cell death nor ferritin cleavage occurred in HCE-T cells incubated with 10 µg/mL of nicotine, which is equivalent to 5% CSE (Fig. 6C,D). Furthermore, NAC protected HCE-T cells against the cell death induced by CSE, but not a high dosage of nicotine (Fig. 6B), suggesting that the mechanism how nicotine induces cell death is different from the one of CSE. The present study revealed that the component(s) other than nicotine of CSE and HTP would damage corneal epithelium through the pathway associated with intracellular iron and ferritin.

## Discussion

**Figure 7:**
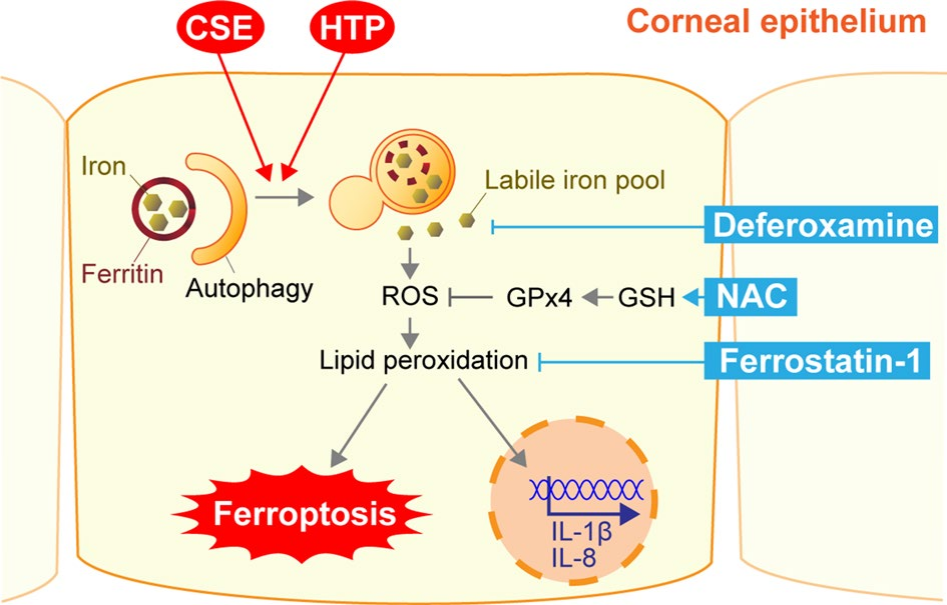
A graphical summary of the results presented in this paper.

In the present study, we analyzed the molecular mechanism that contributes to smoke-induced damage to the corneal epithelium using the HCE-T cell culture model system. CSE exposure perturbed epithelial barrier function and suppressed the expression of mucins (Fig. 1). In addition, alteration of the tight junction structure induced by CSE exposure was markedly restored by NAC treatment (Fig. 1). Both CSE and HTPs independently promoted ferritin degradation (Figs. 3, 5), resulting in the accumulation of bivalent iron ions in autolysosomes, lipid peroxidation, and cell death (Figs. 2, 5, supplementary Fig. 1). A ferroptosis inhibitor, Fer-1, and an iron chelator, DFO, exhibited protective effects against CSE-induced cell damage (Fig. 4). The treatment with nicotine-free HTPs resulted in the cell damage similar to CSE, whereas nicotine alone equivalent CSE did not affect ferritin degradation (Figs. 5, 6). Taken together, we concluded that the ferroptosis signaling pathway is involved in human corneal epithelial cell injury induced by either cigarette smoke or HTPs (Fig. 7).

The ocular surface is one of the tissues directly exposed to the sidestream smoke rather than received the blood-borne toxicants derived from the mainstream smoke. Due to its avascular nature, the corneal epithelium is mostly affected by the sidestream constituents absorbed into the tear film. In the system carried out in the present study, it is assumed that cells would be exposed to an aqueous extract of cigarette smoke at a higher level than the smoke exposure to cornea in situ. Further investigation is necessary to apply our findings to the in-vivo exposure situation of a smoker by using a more adequate model with the more relevant exposure mode (e.g., exposure to an air–liquid interface culture). Meanwhile, the heat-not-burn tobacco system causes less air-pollution than conventional tobacco because it does not generate the sidestream aerosol. However, the exhalated breath contain residual tobacco smoke^15^, which can be a source for secondhand smoke (also called environmental tobacco smoke), suggesting that HTPs are also able to reach to the ocular surface indirectly. Whereas, it should be noted that the effects observed in this study do not necessarily link the environmental HTP exposure to a risk for corneal epithelial disorders such as dry eye diseases at the moment. Further study using an appropriate model system is needed to understand how HTPs affects ocular health.

The nicotine concentration of CSE and HTP used in the present study are 198 µg/mL and 34.8 µg/mL, respectively (Fig. 6A). The concentration of nicotine in blood is average 22 ng/mL in heavy smokers^16^, suggesting that the nicotine amount used in this study would be relatively higher than actual human exposure. Further study such as a murine smoke exposure model is necessary to demonstrate the effect of CSE on the intracellular iron pathway in-vivo. Cigarette smoke contains abundant cytotoxic materials including tar, nicotine, and carbon monoxide^6^. In the manufacturing process of harvested tobacco leaves (i.e., fermentation) nicotine is converted into carcinogenic compounds, including tobacco-specific nitrosamines (TSNAs). Contrary to the fact that mainstream smoke from heated tobacco contains much less carbon monoxide and TSNAs^5^, our results showed that the toxicity of HTPs and NF-HTPs was similar to CSE toxicity (Fig. 5). These results indicated that carbon monoxide, TSNAs, and nicotine do not trigger ferroptosis in cells exposed to CSE, HTPs, or NF-HTPs. Further investigation is necessary to identify the substances(s) that induce ferroptosis in cells exposed to CSE, HTPs, or NF-HTPs. Since the levels of iron derived from mainstream cigarette smoke is too low to cause iron accumulation in the lungs^8^, CSE-initiated iron accumulation and cell death may arise from defects in intracellular iron metabolism via ferritinophagy rather than by an increase in iron uptake.

Corneal iron accumulation can present as iron lines (also referred to as Hudson-Stahli lines) in the normal aging cornea^17^. The malfunction of transferrin contributes to the risk of keratoconus, a progressive thinning corneal dystrophy which is also characterized by corneal iron lines^18^. It is known that iron also contributes to the progression of retinal degeneration^19^. Moreover, iron accumulation in the retina has been found in the eyes of patients with age-related macular degeneration^20^. We have previously reported that chronic light exposure increased the intracellular ferrous iron levels in murine photoreceptor-derived 661W cells^21^. Recently, single-cell RNA sequencing of cells derived from human retinas revealed subpopulations of Müller glia and astrocytes that expressed higher levels of ferritin^22^. Importantly, Müller glial cells play a pivotal role in retina iron homeostasis^23^. Our findings support the idea that iron homeostasis mediated by ferritin plays a key role in the pathogenesis of various ocular diseases.

Exposure to CSE or HTPs enhanced p62 expression (Fig. 5I,K). Autophagy inhibitors further stimulated p62 expression, which suggested that transcriptional up-regulation contributed to the increase in p62 expression (Fig. 3C,E). LC3 expression was also consistently increased, albeit slightly (Fig. 5I). Since p62 regulates the nuclear transport of Nrf2^24^, we monitored the subcellular localization of Nrf2 after exposure to CSE. We observed an increase in Nrf2 expression in the nuclear fraction after exposure to CSE (Fig. 4G), which suggested that CSE activated both the Nrf2 and autophagy pathways. Nrf2 up-regulation contributed to RPE cell survival after exposure to CSE^12^. Moreover, Nrf2 activation attenuated light-induced photoreceptor degeneration by up-regulating the antioxidative enzyme, heme oxygenase-1^25^. On the other hand, pharmacological Nrf2 activation exhibited cytoprotective effects via transiently accelerating autophagy in retinal pigment epithelium cells^26^. Further investigation is necessary to understand the relationship between the Nrf2 signaling pathway and autophagy as well as their role(s) in the corneal epithelial cellular defense against CSE-induced injury.

The GSH precursor NAC has been clinically used to treat dry eye syndrome^27^. We demonstrated that NAC considerably attenuated corneal cell damage induced by either CSE or HTPs (Figs. 1, 5). In addition, depletion of GSH, a potent antioxidant, activated ferroptosis through intracellular lipid peroxidation mediated by glutathione peroxidase (GPx) 4^9^. A previous report showed that overexpression of GPx4 attenuated cigarette smoke-induced pulmonary injury in a murine model^10^. On the other hand, GPx4 deficiency delays wound healing in the corneal epithelium^28^. These findings suggested that antioxidant enzymes associated with ferroptosis may be utilized as novel therapeutic targets for preserving the corneal epithelium.

The corneal epithelium comprises a physiologic barrier between the external and intraocular environments and the tight junction contributes to this function. We demonstrated that CSE treatment disrupted intercellular adhesion (Fig. 1). It was previously reported that sidestream whole smoke inhibited actin reorganization and inactivated both focal adhesion complex molecules as well as Rho-GTPases in the cornea^29^. Interestingly, CSE treatment suppressed the expression of MUC16 (Fig. 1H), which can interact with the actin cytoskeleton via the ezrin/radixin/moesin actin-binding proteins^1^. Thus, cigarette smoke may interrupt the establishment of the apical microenvironment formed by the cytoskeleton and the membrane-associated mucins, which would not only destabilize the tear film but also increase the susceptibility to infection.

We showed that IL-8 expression was up-regulated after exposure to CSE (Fig. 4J). It was also reported that IL-8 levels were increased in the bronchoalveolar lavages of healthy smokers and COPD patients^8^. IL-8 is known to induce corneal neovascularization^30^. Moreover, IL-8 overexpression in the cornea induced angiogenesis and ulcer formation^31^, which suggested that chronic exposure to cigarette smoke may induce aberrant inflammation via increased cytokine production. We have not determined how CSE exposure leads to the up-regulation of cytokine production. The expression of IL-8 is regulated by both IL-1α and Tumor necrosis factor (TNF)-α^32^. Further studies are required to elucidate the signaling pathway that contributes to smoking-induced inflammation in the corneal epithelium.

In conclusion, our studies indicated that iron plays a pivotal role in smoking-induced corneal epithelium damage via aberrant autophagic processing of ferritin. Ferroptosis may be utilized as a new therapeutic target for developing drugs to treat corneal disorders.

## Materials and methods

### Reagents

Primary antibodies directed against the following proteins were utilized: ZO-1 (rabbit, Invitrogen, Carlsbad, CA, USA, Cat# 40-2200, 1:2,000 for immunofluorescence staining; IF), ferritin (rabbit, Abcam, Cambridge, UK, Cat# ab75973, 1:1,000 for immunoblotting; IB, 1:50 for IF), β-actin (mouse, Sigma-Aldrich, St. Louis, MO, USA, Cat# A2228, 1:100,000 for IB), p62/ Sequestosome 1 (SQSTM1; mouse, GeneTex Inc., Irvine, CA, USA, Cat# GTX629890, 1:2,000 for IB, 1:200 for IF), microtubule-associated protein light chain 3 (LC3, rabbit, GeneTex Inc., Cat# GTX127375, 1:10,000 for IB), Nrf2 (rabbit, Santa Cruz Biotechnology Inc., Santa Cruz, CA, USA, Cat# sc-13032, 1:2,000 for IB), c-Jun (rabbit, Cell Signaling Technology, Danvers, MA, USA, Cat# 9165S, 1:10,000 for IB), and GAPDH (rabbit, Cell Signaling Technology, Cat# 2118S, 1:100,000 for IB). The following secondary antibodies were utilized: Alexa Fluor 488 goat anti-rabbit antibody (1:400 for IF), Alexa Fluor 633 goat anti-mouse antibody (1:400 for IF), and horseradish peroxidase (HRP)-conjugated antibody (goat anti-rabbit or goat anti-mouse, 1:10,000 for IB). All the secondary antibodies were purchased from Thermo Fisher Scientific (Waltham, MA, USA). NAC and CQ were purchased from FUJIFILM-Wako (Osaka, Japan). BafA1 was purchased from Cayman Chemical (Ann Arbor, MI, USA). (-)-Nicotine (Cat# N3876), FITC-dextran (2,000 kDa; Cat# FD2000S), DFO (Cat# D9513), and Fer-1 (Cat# SML0583) were purchased from Sigma-Aldrich. Si-RhoNox-1 was a kind gift from Dr. Hideko Nagasawa and Dr. Tasuku Hirayama (Gifu Pharmaceutical University, Gifu, Japan).

### Preparation of CSE and HTPs

Cigarettes were purchased from Japan Tobacco Inc. (Tokyo, Japan). CSE was prepared as described previously with minor modifications^33^. Briefly, cigarette smoke from one cigarette was collected from the side of the combustion zone with neither puffing nor passing through a filter, and bubbled into 10 mL of sterile PBS in a 50 mL centrifuge tube at minimum speed using a suction pump (Tokyo M.I., Tokyo, Japan, Cat# M20). The CSE solution was then filtered with a 0.22 µm polyvinylidene fluoride (PVDF) membrane filter (Merck Millipore, Darmstadt, Germany, Cat# SLGVR33RS) to remove insoluble particles. This 100% CSE solution was then utilized in the experiments described below.

The heated tobacco and heat-stick device were purchased from Philip Morris International Inc. (New York, NY, USA). To generate an aerosol, one heat stick was inserted into the heat-stick device, and the other side of the stick was connected to M20 suction pump via a silicone tube (8 mm inner diameter; 60 cm long). The heater was actuated by air-suction at minimum speed during the operation. The aerosol generated from one heat stick was bubbled into 10 mL of sterile PBS. The HTP solution was filtered with a 0.22 µm PVDF membrane filter, then used in the following experiments. Nicotine-free heat sticks were purchased from VUEN Co., Ltd. (Osaka, Japan), and NF-HTPs were extracted in the same way as HTPs.

Nicotine concentrations were quantified by Shimadzu Techno-Research, Inc. (Kyoto, Japan) using a high-performance liquid chromatography (HPLC) equipped with CERI L-Column2 ODS 5 µm (150 mm × 4.6 mm i.d.) and SPD-M20A (Shimadzu, Kyoto, Japan).

### Cell culture

HCE-T cells^34^ were obtained from the RIKEN BioResource Center Cell Bank (Ibaraki, Japan) and maintained as described previously^35^. The cells were grown at 37 °C in a humidified atmosphere of 5% CO_2_ and passaged by trypsinization every 2 or 3 days.

### Cell death analyses and cell viability assays

Cell death analyses and cell viability assays were performed as described previously^35^. The images were collected using a Lionheart FX Automated Microscope (Bio Tek Instruments, Winooski, VT, USA) and the percentage of PI-positive cells was automatically calculated as a cell death ratio by the Gen5 software (Bio Tek Instruments). Cell viability assays were conducted using the Cell Counting Kit-8 (CCK-8) according to the manufacturer's instructions (Dojindo Laboratories, Kumamoto, Japan). At 0 h and 2 h time points, the absorbance was measured at 450 nm using the Varioskan Flash 2.4 microplate reader (Thermo Fisher Scientific).

### TER measurements

Epithelial barrier function was evaluated by TER analyses. HCE-T cells were seeded at a density of 5,000 cells per well into Transwell plates containing a 0.4 μm pore polyester membrane insert (Corning Inc., Cat# 3470). After 24 h, the medium was replaced with fresh DMEM/F-12 medium (FUJIFILM-Wako, Osaka, Japan) containing 1% FBS with or without either 5% CSE or 5% CSE plus 1 mM NAC. The TER values were measured every 24 h until harvest using an epithelial volt-ohm meter (Millicell ERS-2; Merck Millipore) and a cup-shaped electrode (Endohm-6; World Precision Instruments, Sarasota, FL, USA).

### Immunostaining of polarized cells on Transwell plates

Immunostaining was performed at day 6 after CSE exposure as described previously with minor modifications^36^. Briefly, cells cultured on a Transwell membrane were washed with PBS containing 0.2 mM CaCl_2_ and 2 mM MgCl_2_ (PBS-C/M), followed by fixation with 4% paraformaldehyde (Electron Microscopy Sciences, Fort Washington, PA, USA) in PBS-C/M for 10 min at room temperature. The membranes were enucleated using a sharp blade, transferred onto a sheet of parafilm in a humidified tray, and then quenched with 50 mM NH_4_Cl in PBS-C/M for 10 min at room temperature. After incubation with blocking buffer (PBS-C/M containing 0.5% BSA, 0.5% Triton X-100, and 0.2 mg/ml sodium azide) for 30 min at room temperature, the membranes were incubated with anti-ZO-1 antibody in PBS-C/M for 1 h at room temperature. Next, the membranes were incubated with Alexa Fluor 488 secondary antibody in PBS-C/M containing 16.2 µM Hoechst 33342 (Thermo Fisher Scientific) for 30 min at room temperature. After three washes with PBS-C/M for 5 min, the samples were mounted onto a slide with ProLong Gold antifade reagent (Thermo Fisher Scientific). The images were acquired using a 40 × objective lens on an Olympus FLUOVIEW FV3000 confocal laser scanning microscope (Olympus Co., Tokyo, Japan). The cell area and circumference were obtained from the projected images using ImageJ software (National Institutes of Health, Bethesda, MD, USA). For each experiment, 100 cells were analyzed by the third person in a blind fashion. For presentation purposes, the images were edited using ImageJ or Adobe Photoshop CC2019 software (Adobe, San Francisco, CA, USA).

### Quantitative RT-PCR (qRT-PCR)

qRT-PCR was performed according to the manufacturer’s instructions. Briefly, total RNA was purified from HCE-T cells using the NucleoSpin RNA kit (Takara Bio, Shiga, Japan). A cDNA library was generated using the PrimeScript RT Reagent Kit (Takara Bio). Quantitative PCR was conducted with the TB Green Premix Ex Taq II Reagent (Takara Bio) on a Thermal Cycler Dice Real Time System III (Takara Bio). The primer pairs were designed as described previously^37,38^. The C_T_ values were normalized to the GAPDH C_T_ values and are expressed as fold increases in gene expression.

### BODIPY 581/591 C11 Lipid Peroxidation Sensor

The BODIPY 581/591 C11 (Thermo Fisher Scientific) Lipid Peroxidation Sensor assay was performed according to the manufacturer's instructions. Briefly, cells were incubated with 10 µM BODIPY 581/591 C11 for 30 min at 37 °C. For live imaging, the medium was replaced with live imaging buffer [Live Cell Imaging Solution (LCIS, Thermo Fisher Scientific) supplemented with 4.5 g/L glucose and 1% FBS], and then recorded with a 10 × objective lens on a Lionheart FX Automated Microscope (Bio Tek Instruments). The image analyses were performed with Gen5 software (Bio Tek Instruments).

### Cell lysis and immunoblotting

HCE-T cells cultured in 12-well plates (Corning Inc.) were harvested as described previously^39^. The lysates (2 µg of protein per lane) in RIPA buffer (Sigma-Aldrich, Cat# R0278) containing a protease inhibitor cocktail as well as phosphatase inhibitor cocktails 2 and 3 (Sigma-Aldrich) were subjected to SDS-PAGE on 5–20% gradient gels (SuperSep Ace, FUJIFILM-Wako) followed by immunoblotting.

### Live imaging of intracellular bivalent iron and FITC-dextran uptake

Intracellular ferrous ion [Fe(II)] distribution was evaluated by Si-RhoNox-1 staining as described previously^40^. Briefly, cells grown on a glass bottom dish (CELLview, Greiner Bio-One, Frickenhausen, Germany) were cultured with or without 5% CSE for 24 h. Next, the cells were rinsed once with pre-warmed live imaging buffer and then incubated with 5 µM Si-RhoNox-1 and 16.2 µM Hoechst 33342 in live imaging buffer for 30 min at 37 °C. After replacing the medium with fresh live imaging buffer, images were acquired using a 40 × objective lens on an Olympus FLUOVIEW FV3000 confocal laser scanning microscope (Olympus Co., Tokyo, Japan). In order to visualize autolysosomal compartments, FITC-dextran (0.5 mg/ml) was added to the medium of cells that were treated with 5% CSE. These cells were then subjected to Si-RhoNox-1 staining as described above.

### Labeling lysosomes with LysoTracker Red and immunostaining

In order to visualize acidic compartments, cells were stained with LysoTracker Red DND-99 (Thermo Fisher Scientific) according to the manufacturer's instructions. Briefly, cells grown on 12 mm glass coverslips (Matsunami Glass Industry, Osaka, Japan, Cat# 1-S) coated with 0.2% gelatin from porcine skin (Sigma Aldrich, Cat# G2500) were incubated with 75 nM LysoTracker Red for 30 min at 37 °C in the dark. Next, the cells were rinsed once with PBS-C/M and fixed with 4% paraformaldehyde in PBS-C/M for 10 min at room temperature. The fixed samples were then quenched with 50 mM NH_4_Cl in PBS-C/M for 10 min, followed by blocking with PBS-C/M containing 0.5% BSA, 0.5% saponin, and 0.2 mg/ml sodium azide for 30 min. The samples were then subjected to immunostaining as described above. The images were acquired with a 40 × objective lens on a Zeiss LSM700 microscope (Carl Zeiss, Germany).

### Nuclear protein extraction

HCE-T cells cultured in 60 mm dishes (Corning Inc.) were rinsed twice with ice-cold PBS and then harvested by scraping in the presence of 500 µl of hypotonic buffer [20 mM Tris–HCl (pH 7.4), 10 mM NaCl, and 3 mM MgCl_2_] containing a protease inhibitor cocktail as well as phosphatase inhibitor cocktails 2 and 3 (Sigma-Aldrich). After incubation on ice for 15 min, 25 µl of 10% (v/v) Nonidet P-40 (Nacalai Tesque, Kyoto, Japan) was added to the cell suspension, followed by vortexing for 10 s at maximum speed. Next, the samples were centrifuged at 3,000 rpm for 10 min at 4 °C in an AF-2724 rotor (Kubota, Tokyo, Japan). The supernatant was collected as the cytoplasmic fraction. The pellet was rinsed once with hypotonic buffer and lysed by vigorous vortexing in 50 µl of RIPA buffer containing a protease inhibitor cocktail as well as phosphatase inhibitor cocktails 2 and 3. The lysate was centrifuged at 12,000 × *g* for 20 min at 4 °C, and the supernatant was collected as the nuclear fraction. The samples were boiled with sample buffer solution (FUJIFILM-Wako) for 5 min, and then subjected to immunoblotting as described above.

### Statistics

The data are represented as the mean ± standard error of the mean (SEM). The statistical analyses were performed with either the Student’s *t*-test or Dunnett’s test using SPSS Statistical software (IBM, Armonk, NY, USA).

## Supplementary Information

Supplementary Information.

## References

[1] Blalock TD (2007). Functions of MUC16 in corneal epithelial cells. Invest. Ophthalmol. Vis. Sci..

[2] Altinors DD (2006). Smoking associated with damage to the lipid layer of the ocular surface. Am. J. Ophthalmol..

[3] Higuchi A (2011). Corneal damage and lacrimal gland dysfunction in a smoking rat model. Free Radic. Biol. Med..

[4] Thornton J (2005). Smoking and age-related macular degeneration: a review of association. Eye.

[5] Bekki K, Inaba Y, Uchiyama S, Kunugita N (2017). Comparison of Chemicals in Mainstream Smoke in Heat-not-burn Tobacco and Combustion Cigarettes. J. UOEH.

[6] Rodgman A, Perfetti TA (2016). The Chemical Components of Tobacco and Tobacco Smoke.

[7] De Oca MM (2017). Smoke, biomass exposure, and COPD risk in the primary care setting: the PUMA study. Respir. Care.

[8] Ghio AJ (2008). Particulate matter in cigarette smoke alters iron homeostasis to produce a biological effect. Am. J. Respir. Crit. Care Med..

[9] Dixon SJ (2012). Ferroptosis: An iron-dependent form of nonapoptotic cell death. Cell.

[10] Yoshida M (2019). Involvement of cigarette smoke-induced epithelial cell ferroptosis in COPD pathogenesis. Nat. Commun..

[11] Mancias JD, Wang X, Gygi SP, Harper JW, Kimmelman AC (2014). Quantitative proteomics identifies NCOA4 as the cargo receptor mediating ferritinophagy. Nature.

[12] Huang C (2015). Activation of the UPR protects against cigarette smoke-induced RPE apoptosis through up-regulation of Nrf2. J. Biol. Chem..

[13] Su L-J (2019). Reactive oxygen species-induced lipid peroxidation in apoptosis, autophagy, and ferroptosis. Oxid. Med. Cell. Longev..

[14] Hirayama T, Okuda K, Nagasawa H (2013). A highly selective turn-on fluorescent probe for iron(ii) to visualize labile iron in living cells. Chem. Sci..

[15] Invernizzi G, Ruprecht A, De Marco C, Paredi P, Boffi R (2007). Residual tobacco smoke: Measurement of its washout time in the lung and of its contribution to environmental tobacco smoke. Tob. Control.

[16] Benowitz NL, Kuyt F, Jacob P, Jones RT, Osman AL (1983). Cotinine disposition and effects. Clin. Pharmacol. Ther..

[17] Loh A, Hadziahmetovic M, Dunaief JL (2009). Iron homeostasis and eye disease. Biochim. Biophys. Acta.

[18] Wójcik KA (2013). Polymorphism of the transferrin gene in eye diseases: Keratoconus and Fuchs endothelial corneal dystrophy. Biomed. Res. Int..

[19] He X (2007). Iron homeostasis and toxicity in retinal degeneration. Prog. Retin. Eye Res..

[20] Song D, Dunaief JL (2013). Retinal iron homeostasis in health and disease. Front. Aging Neurosci..

[21] Imamura T (2014). Hydroxyl radicals cause fluctuation in intracellular ferrous ion levels upon light exposure during photoreceptor cell death. Exp. Eye Res..

[22] Menon M (2019). Single-cell transcriptomic atlas of the human retina identifies cell types associated with age-related macular degeneration. Nat. Commun..

[23] Baumann B (2017). Conditional Müller cell ablation leads to retinal iron accumulation. Invest. Ophthalmol. Vis. Sci..

[24] Jiang T (2015). p62 links autophagy and Nrf2 signaling. Free Radic. Biol. Med..

[25] Inoue Y (2017). RS9, a novel Nrf2 activator, attenuates light-induced death of cells of photoreceptor cells and Müller glia cells. J. Neurochem..

[26] Saito Y (2018). Transient acceleration of autophagic degradation by pharmacological Nrf2 activation is important for retinal pigment epithelium cell survival. Redox Biol.

[27] Haut J, Labrune P, Ullern M, Chermet M (1977). New trial treatment of dry eye with acetylcysteine ophthalmic solution. Bull. Soc. Ophtalmol. Fr..

[28] Sakai O, Uchida T, Imai H, Ueta T (2016). Glutathione peroxidase 4 plays an important role in oxidative homeostasis and wound repair in corneal epithelial cells. FEBS Open Bio.

[29] Yuan H, Ma C, Moinet L, Sato N, Martins-Green M (2010). Reversal of second-hand cigarette smoke-induced impairment of corneal wound healing by thymosin beta4 combined with anti-inflammatory agents. Invest. Ophthalmol. Vis. Sci..

[30] Strieter RM (1992). Interleukin-8. A corneal factor that induces neovascularization. Am. J. Pathol..

[31] Oka M, Norose K, Matsushima K, Nishigori C, Herlyn M (2006). Overexpression of IL-8 in the cornea induces ulcer formation in the SCID mouse. Br. J. Ophthalmol..

[32] Cubitt CL, Tang Q, Monteiro CA, Lausch RN, Oakes JE (1993). IL-8 gene expression in cultures of human corneal epithelial cells and keratocytes. Invest. Ophthalmol. Vis. Sci..

[33] Fujii S (2012). Insufficient autophagy promotes bronchial epithelial cell senescence in chronic obstructive pulmonary disease. Oncoimmunology.

[34] Araki-Sasaki K (1995). An SV40-immortalized human corneal epithelial cell line and its characterization. Invest. Ophthalmol. Vis. Sci..

[35] Ishida K (2021). Free-Radical Scavenger NSP-116 Protects the Corneal Epithelium against UV-A and Blue LED Light Exposure. Biol. Pharm. Bull..

[36] Otsu W, Hsu Y-C, Chuang J-Z, Sung C-H (2019). The late endosomal pathway regulates the ciliary targeting of tetraspanin protein peripherin 2. J. Neurosci..

[37] Imayasu M, Hori Y, DwightCavanagh H (2010). Effects of multipurpose contact lens care solutions and their ingredients on membrane-associated mucins of human corneal epithelial cells. Eye Contact Lens.

[38] Lee SH, Kim KW, Joo K, Kim JC (2016). Angiogenin ameliorates corneal opacity and neovascularization via regulating immune response in corneal fibroblasts. BMC Ophthalmol..

[39] Otsu W (2020). Blue light-emitting diode irradiation promotes transcription factor EB-mediated lysosome biogenesis and lysosomal cell death in murine photoreceptor-derived cells. Biochem. Biophys. Res. Commun..

[40] Imai T (2019). Intracellular Fe 2+ accumulation in endothelial cells and pericytes induces blood-brain barrier dysfunction in secondary brain injury after brain hemorrhage. Sci. Rep..

