# Supplementary material for: Cigarette smoke extract and heated tobacco products promote ferritin cleavage and iron accumulation in human corneal epithelial cells
Source: Sci Rep. 2021 Sep 17;11:18555. doi: 10.1038/s41598-021-97956-3 (PMC8448754; doi:10.1038/s41598-021-97956-3)
Supplement: Supplementary file 1 — Supplementary Information. [file 41598_2021_97956_MOESM1_ESM.pdf]

Supplementary information

Cigarette smoke extract and heated tobacco products promote ferritin cleavage and iron accumulation in human corneal epithelial cells

Wataru Otsu[1,#], Kodai Ishida[1,#], Naoki Chinen[2], Shinsuke Nakamura[2], Masamitsu Shimazawa[1,2], Hideshi Tsusaki[1], and Hideaki Hara[1,2,*]

[1]Department of Biomedical Research Laboratory, [2]Molecular Pharmacology, Department of Biofunctional Evaluation, Gifu Pharmaceutical University, 1-25-4 Daigaku-nishi, Gifu, 501-1196, Japan

[#]These authors contributed equally.

***Corresponding Author:**

Hideaki Hara, Ph.D., R.Ph.,

Molecular Pharmacology, Department of Biofunctional Evaluation, Gifu Pharmaceutical University, 1-25-4 Daigaku-nishi, Gifu, 501-1196, Japan;

E-mail: hidehara@gifu-pu.ac.jp;

Tel and Fax: +81-58-230-8126

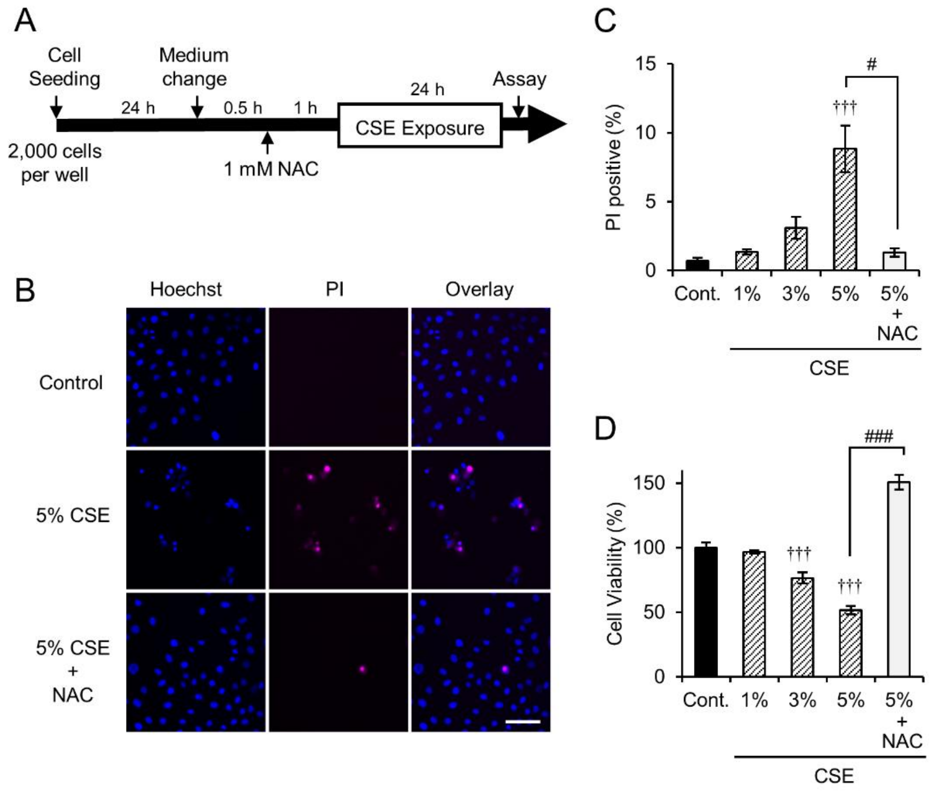

Supplementary Figure 1. NAC treatment protected HCE-T cells from CSE-induced cell death. (A) The time course of NAC treatment and CSE exposure of HCE-T cells. (B) Representative images of PI (magenta) and Hoechst 33342 (blue) staining after CSE exposure in the presence or absence of NAC. Bar = 100 µm. (C) The bars show the PI-positive cell ratios. (D) Cell viability was measured with a CCK-8 assay. The data are represented as the mean ± SEM (n = 6). $^{†††}P < 0.001$, Dunnett's test vs. Control (Cont); $^{\#}P < 0.05$, $^{\#\#\#}P < 0.001$, Student's *t*-test vs. 5% CSE.

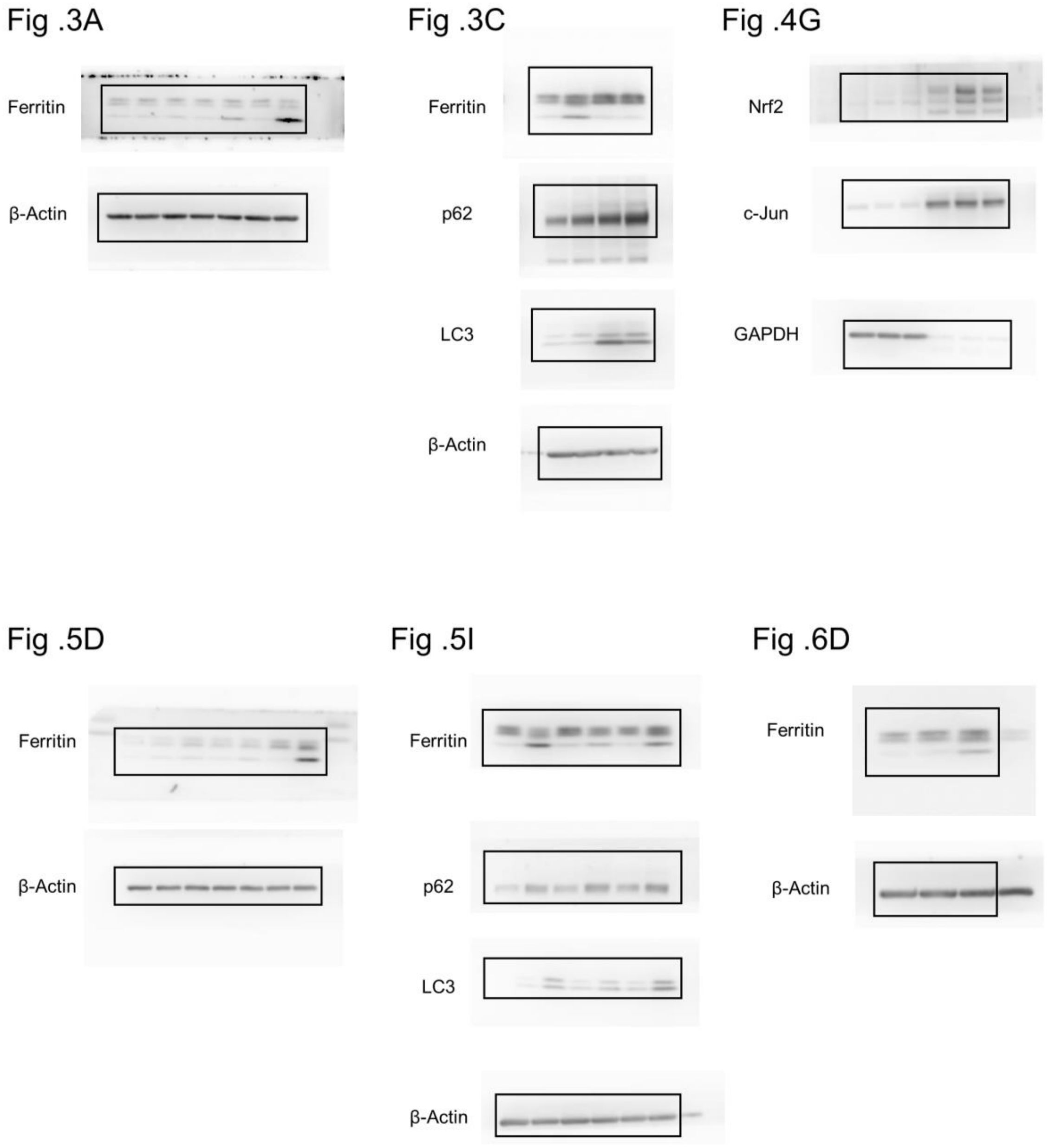

Supplementary Figure 2. Full scans of immunoblots. The boxed regions are presented in the indicated figures.
